# Evaluation of Serum Electrolyte Levels in Patients With Anemia

**DOI:** 10.7759/cureus.18417

**Published:** 2021-10-01

**Authors:** Farah Mansoor, Pinky Bai, Navneet Kaur, Sandresh Sultan, Sucheta Sharma, Anum Dilip, Yasir Kammawal, Simra Shahid, Amber Rizwan

**Affiliations:** 1 Internal Medicine, Jinnah Sindh Medical University, Karachi, PAK; 2 Internal Medicine, Adesh Institute of Medical Sciences and Research, Buchu Kalan, IND; 3 Internal Medicine, Jinnah Post Graduate Medical Centre, Karachi, PAK; 4 Internal Medicine, Punjab Institute of Medical Sciences, Jalandhar, IND; 5 Anatomical and Clinical Pathology, University of Arizona College of Medicine-Tucson, Tucson, USA; 6 Family Medicine, Jinnah Post Graduate Medical Center, Karachi, PAK

**Keywords:** serum electrolytes, na-k atpase, electrolyte imbalance, anemia, iron deficiency anemia (ida)

## Abstract

Introduction: Anemia is one of the most prevalent diseases globally. Various diseases have linked anemia with electrolyte disturbance. However, the local data are limited. In this study, we will determine the prevalence of electrolyte imbalance in anemic patients.

Methods: This case-control study was conducted in a tertiary care hospital from January 2021 to July 2021. A total of 500 anemic patients were enrolled in the study after informed consent. Another 500 non-anemic patients were enrolled as the control group. Blood was taken from both groups and send for assessment of electrolytes.

Results: Sodium levels were significantly lower in anemic patients compared to non-anemic patients (131.42 ± 0.82 meq/L vs. 135.57 ± 0.42 meq/L; p-value: <0.0001). Potassium levels were significantly higher in anemic patients compared to non-anemic participants (4.37 ± 0.12 meq/L vs. 4.09 ± 0.11 meq/L; p-value: <0.0001). Chloride levels were significantly higher in participants with anemia compared to non-anemic participants (103.92 ± 0.46 meq/L vs. 100.99 ± 0.41 meq/L).

Conclusion: Our study indicates that sodium levels and potassium levels are impacted in patients with anemia compared to patients without anemia. Close monitoring of serum electrolytes is suggested in patients with anemia to avoid complications and life-threatening conditions.

## Introduction

It is estimated that one-third of the global population is anemic, affecting more than two billion people worldwide with mortality of almost 800,000 per year [1]. Common manifestations are fatigue, dyspnea, pallor, delayed growth and development in neonates, learning and behavioral abnormalities in adolescents, and restless leg syndrome in adults. Iron deficiency anemia (IDA) is the most prevalent type of anemia with an annual incidence rate of 7.2-13.96 per 1000 people per year. It is a laboratorial diagnosis with a low ferritin level or low percent transferrin saturation with an increased iron-binding capacity [2]. IDA is the world’s most widespread nutritional disorder, affecting both industrialized and developing countries, irrespective of gender, or socioeconomic status, with dietary iron deficiency being the most common cause [3]. Risk is high during adolescence as a growth spurt, pubertal developments, and physical activity increase the demand [4].

Few studies have strongly associated anemia with an imbalance in the serum electrolyte levels due to alteration in red cell membrane-bound sodium-potassium adenosine triphosphatase (Na+/K+ ATPase) pump activity that regulates intra- and extracellular cation homeostasis. The serum electrolytes, i.e., sodium (Na+), potassium (K^+^), chloride (Cl^−^), and bicarbonate ($\mathrm{HCO_{3}^{-}}$) are essential for maintaining the normal shape of red blood cells (RBCs), and are responsible for the exchange of gases between RBCs and tissues, also plays an essential role in nerve conduction, muscle contraction and acid-base balance [5]. Elevation of Na^+^/K^+^ ATPase activity in anemic patients compensates for the mechanism for adaptation of the patients with low oxygen and its physiological role in the cell, change in membrane-bound enzymes directly affects the Na^+^ and K^+^ in the serum [6,7].

According to recent studies, iron-deficient patients have low levels of sodium and bicarbonates while potassium and chloride were high. Similarly, low levels of sodium and high potassium and chloride levels were found in patients with sickle cell anemia. Alteration in serum electrolyte levels can cause mild symptoms like lethargy, fatigue, muscle cramping to severe symptoms including irregular heartbeat, confusion, convulsion, and even death [8]. Since both anemia and serum electrolyte imbalances are the two most significantly prevalent public health issues, the suggested interlink between the two holds great clinical importance [7]. Global data show a relationship between anemia, particularly IDA, with electrolyte imbalance. However, the local data are limited. In this study, we will determine the prevalence of electrolyte imbalance in anemia. This study will bring out the current data and encourage the physicians for a broader approach towards anemic patients.

## Materials and methods

This case-control study was conducted in a tertiary care hospital in the internal medicine unit, from January 2021 to July 2021. A total of 500 anemic patients were enrolled in the study after obtaining informed consent. Another 500 non-anemic patients were also enrolled as the control group. The ethical review board approval was taken from Jinnah Sindh Medical University (JSMU/IRB/2021/16) before participants’ enrollment. Participants were enrolled via consecutive convenient non-probability sampling. Anemia was confirmed via a complete blood count report. Anemia is defined as hemoglobin levels <12.0 g/dL in women and <13.0 g/dL in men [9]. Participants with chronic kidney diseases, malignancy, and infections were excluded from the study, to reduce the risk of including participants with electrolyte disturbance due to other diseases.

After enrollment, 5 mL of blood was drawn from the cubital vein via phlebotomy and sent to the laboratory for assessment of electrolytes. Statistical Package for Social Sciences, version 21.0 (SPSS, IBM Corporation, Armonk, New York, United States) was used to analyze the data. Continuous variables were tabulated as mean and standard deviation, while categorical data were presented as percentages and frequencies. Mean electrolyte values were compared using an independent t-test. A p-value of less than 0.05 was considered significant and the null hypothesis was void.

## Results

The mean age in both case and control groups was compared and no significant difference was found (48 ± 7 years vs. 47 ± 7 years). The number of female participants were more than the male participants; however, the ratio was consistent in both case and control group (69.9% vs. 68.4%). Other demographics are defined in Table 1.

**Table 1:** Demographics of study participants NS: nonsignificant

Demographics	Case group (n=500)	Control group (n=500)	p-value
Mean age (in years)	48 ± 7	47 ± 7	NS
Gender			
Male	152 (30.4%)	158 (31.6%)	NS
Female	348 (69.6%)	342 (68.4%)	NS
Smokers (%)	101 (20.2%)	99 (19.8%)	NS
Type 2 diabetes (%)	92 (18.4%)	90 (18.0%)	NS
Hypertension (%)	102 (20.4%)	109 (21.8%)	NS

Na^+^ levels were significantly lower in anemic patients compared to non-anemic patients (131.42 ± 0.82 meq/L vs. 135.57 ± 0.42 meq/L; p-value: <0.0001). K^+^ levels were significantly higher in anemic patients compared to non-anemic participants (4.37 ± 0.12 meq/L vs. 4.09 ± 0.11 meq/L; p-value: <0.0001). Cl^−^ levels were significantly higher in participants with anemia compared to non-anemic participants (103.92±0.46 meq/L vs. 100.99±0.41 meq/L; Table 2).

**Table 2:** Comparison of serum electrolyte levels in case and control groups Cl−: chloride, $\mathrm{HCO_{3}^{-}}$: bicarbonate, K^+^: potassium, mEq/L: milliequivalents per liter, Na^+^: sodium, NS: nonsignificant

Electrolytes (meq/L)	Case study (n=500)	Control study (n=500)	p-value
Na^+^	131.42±0.82	135.57±0.42	<0.0001
K^+^	4.37±0.12	4.09±0.11	<0.0001
Cl^−^	103.92±0.46	100.99±0.41	<0.0001
$\mathrm{HCO_{3}^{-}}$	24.10±0.31	24.16±0.34	NS

## Discussion

Iron is important for oxygen supply and utilization in the human body by maintaining the shape of RBC. In this study, it was observed that serum levels of K+ and Cl- were significantly higher in anemic patients when compared with healthy controls. However, it is of interest to note that serum Na^+^ levels were decreased. This is in line with Rafiq et al. which also found that variance in serum electrolyte levels exists among patients with IDA and those without anemia [6]. Antwi-Boasiako et al. also found low Na^+^ levels and high K^+^ and Cl^−^ in patients with sickle cell anemia [10].

For basic life functions, such as generation and conduction of action potential in nerves and muscles, and maintenance of electrical neutrality of cells, electrolytes play a significant role. Calcium, Na^+^, K^+^, Cl^−^, and $\mathrm{HCO_{3}^{-}}$ are important electrolytes. Their levels can be either high or low if an abnormality occurs and this may affect body functions and may lead to life-threatening conditions. It is regarded that hyponatremia is the most frequent electrolyte disorder among electrolyte imbalance conditions. It presents with neurological symptoms, including delirium, nausea, headache, and vomiting. Hyperkalemia, usually associated with cardiac abnormalities, includes arrhythmia and hypochloremia usually occurs due to gastrointestinal losses of $\mathrm{HCO_{3}^{-}}$ ions [11].

Normally, the extracellular environment comprises higher levels of Na^+^ and the intracellular environment contains more K^+^ ions. This environment is maintained by Na^+^/K^+^ ATPase, found on the cell membranes of RBC. It is mainly sensitive to the change in pH, membrane integrity, and volume, and it pumps three Na^+^ ions outside the cell and two K^+^ ions into the cells [12]. Studies concluded the significance of Na^+^/K ATPase as an indicator of blood disorders, including anemia [13]. The literature discussed the generation of compound malonyl dialdehyde, an endproduct of lipid peroxidation when iron deficient RBC was incubated in vitro with hydrogen peroxide. This peroxidation in iron-deficient RBC causes an increase in the membrane stiffness and may, in turn, contribute to impair red cell continued existence [14]. Hence, the peroxidation factor might be responsible for serum electrolyte abnormality, where a change in membrane dynamics and permeability can affect the Na^+^/K^+^ pump. In another study, polycarbonate filters were used to study the rheological properties of erythrocytes in patients with IDA. It concluded that the ratio between the cell surface and cell volume is unfavorable in IDA; hence, RBC can be prematurely sequestered during its passage through the spleen [15]. This might be yet another factor responsible for Na^+^/K^+^ pump impairment, thus, leading to electrolyte imbalance in IDA.

The activity of Na^+^/K^+^ ATPase is enhanced in sickle cell disease, contributing to cellular dehydration [16]. In sickle cell anemia, reduced Na^+^ levels are possibly due to the loss of some body fluids, as well as inflammation [17], whereas higher K^+^ levels are observed secondary to dehydration and sickling of cells, causing a shift of potassium to extracellular space [18].

Common symptoms of electrolyte imbalance include irregular heartbeat, irregular bowel movements, fatigue, and numbness [19]. Therefore, electrolyte imbalance in IDA might be associated with subsequent complications if appropriate interventions and periodic monitoring or screening do not take place. It is important to conduct a physical examination, echocardiography, and screen levels of electrolytes in patients with IDA to avoid any imbalance and complications [19].

Iron deficient people taking iron supplements or pills and iron injections to meet the demands of iron might lead to secondary hemochromatosis or iron overload [20]. The iron overload has an effect on the activity of Na^+^/K^−^ ATPase, and the lipid profile of the human RBC membrane. Iron overload causes the peroxidation of lipids by the production of reactive oxygen species which cause alteration in membrane fluidity and the activities of membrane enzymes [20]. This might also create an imbalance between the serum electrolytes where Na^+^/K− pump plays a significant role. Thus, it is significant to have close monitoring of iron-deficient patients for their iron intake along with monitoring of electrolyte levels to evade impediment during the management of these patients [20].

To the best of our knowledge, this is the first study in the regional setting to study the impact of anemia on serum electrolytes. However, our study has its limitation, and hence, care should be taken while inferring the result to a larger population. First, since it was taken from a single institute, participant’s diversity was limited. Second, since it was a case-control study, the definite association between anemia and electrolyte imbalance could not be established.

## Conclusions

Our study indicates that sodium levels and potassium levels are impacted in patients with anemic compared to patients without anemia. Close monitoring of serum electrolytes is suggested in patients with anemia to avoid complications and life-threatening conditions. Further research is needed for the evaluation of other factors responsible for the imbalance of serum electrolytes in anemic patients as it is still a debatable topic in the literature.
